# Exploring the Role of Phytoconstituent From *Euphorbia neriifolia* Targeting IL‐17A in Psoriasis: In Silico and In Vitro Study

**DOI:** 10.1002/fsn3.71352

**Published:** 2025-12-17

**Authors:** Nitin Sharma, Sanjeev Kumar Sahu, Pankaj Verma, Tanmoy Roy, Sourbh Suren Garg, Manish Vyas, Anil Kumar Sah

**Affiliations:** ^1^ Akums Drugs & Pharmaceuticals Ltd Pure & Cure Healthcare Haridwar Uttarakhand India; ^2^ School of Pharmaceutical Sciences Lovely Professional University Phagwara Punjab India; ^3^ School of Chemical Engineering and Physical Sciences Lovely Professional University Phagwara Punjab India; ^4^ School of Bioengineering and Biosciences Lovely Professional University Phagwara Punjab India; ^5^ Research and Development Cell Parul University Waghodia Gujarat India; ^6^ Gorkha Ayurved Company Pvt. Ltd. Gorkha Haramtari Gandaki Nepal

**Keywords:** DFT, *Euphorbia neriifolia*, in silico, in vitro, molecular docking, psoriasis

## Abstract

Psoriasis is a chronic autoimmune skin disorder characterized by persistent inflammation and excessive keratinocyte proliferation. Although conventional immunosuppressive therapies are clinically effective, their long‐term use is often associated with adverse side effects, highlighting the need for safer alternative treatments. *Euphorbia neriifolia* , a traditional medicinal plant known for its anti‐inflammatory properties, has been investigated for its potential anti‐psoriatic activity. In the present study, molecular docking was performed to evaluate the binding affinities of key phytoconstituents from *E. neriifolia* against interleukin‐17A (IL‐17A; PDB ID: 5HI4), a major cytokine implicated in psoriasis pathogenesis. Docking analysis revealed that compounds such as Taraxerol, Glutinol acetate, and Epifriedelanol showed the highest binding affinities, with scores of −10.3, −10.0, and −10.0 kcal/mol, respectively, surpassing the reference drug methotrexate (−9.2 kcal/mol). Density functional theory (DFT) calculations further supported the electronic stability and reactivity of the top‐ranked compounds, indicating their potential suitability as bioactive leads. The integration of docking and DFT results highlights the novelty of this study, as *E. neriifolia* phytochemicals have not been extensively explored against IL‐17A compared to other medicinal plants studied for psoriasis. The in vitro biological activity of the plant extract was also evaluated by using the L929 fibroblast bioassay, and the IC_50_ value was found to be 168.18 μg/mL. Overall, the findings suggest that selected phytochemicals from *E. neriifolia* exhibit promising interactions with IL‐17A, supporting their possible therapeutic role in psoriasis. These findings support further mechanistic and in vivo studies to validate IL‐17A inhibition.

## Graphical abstract

This research explores the various phytoconstituents from *Euphorbia neriifolia* targeting IL‐17A through in silico and in vitro studies for its role in the more precise and effective therapy in Psoriasis. Molecular docking revealed Taraxerol, Glutinol acetate, and Epifriedelanol showed strong IL‐17A binding (−10.3 to −10.0 kcal/mol), outperforming methotrexate (−9.2 kcal/mol). DFT analysis confirmed their stability, and in vitro L929 fibroblast assay (IC_50_ = 168.18 μg/mL) supported bioactivity, highlighting promising therapeutic leads against psoriasis.

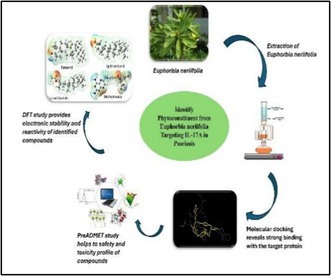

## 1 Introduction

Psoriasis is a chronic, inflammatory, and non‐infectious skin disease characterized by clear identifiable erythematous plaques and globular plaques covered with silver scales (Singh et al. 2013). About 125 million people are affected by psoriasis, which accounts for 2%–3% of the world's total population. As per the World Psoriasis Day Consortium 2023, the Greek word “Psoriasis” means itch, scurf, and rash (Al‐Shobaili and Qureshi 2013). Psoriasis can occur at any age, although there are normally two stages of the disease that begin to occur. Men often get the illness between the ages of 26 and 39, or 60 and 69, whereas women develop it between the ages of 18 and 29 or 50 to 59. Pediatrics also suffer from an increase in prevalence, with 0.14% between 1 and 3 years of age and 0.69% between 14 and 18 years of age, indicating that psoriasis is not common among children (Iskandar et al. 2021). The percentage of global psoriasis published in the countries varies between 0.9%–0.12% (Gibbs 1996) and 12.4% (Danielsen et al. 2013). Psoriasis is therefore an important factor that seriously threatens people's health. Psoriasis mainly affects the nails or skin and is associated with various diseases. Skin injuries are usually primary when they are limited to a region, or secondary when they are more widespread, although they are characterized mainly by uniform, strongly characterized bright red pustules as well as streaks, which are generally whiter than silver or metallic. Symptoms of lesion formation include itching, stinging, and pain (Vena et al. 2010). Psoriatic arthritis, or chronic inflammatory arthritis, is a condition that causes joint deformities and impairment in 1.3% to 34.7% (Pariser et al. 2016) of people with psoriasis. According to evidence, psoriatic individuals are more prone to have extra major clinical issues, such as heart problems as well as communicable illnesses (Boehncke 2018).

Psoriasis is also recognized as a chronic immune‐mediated disorder driven predominantly by the IL‐23/IL‐17 cascade. It acts as a master effector cytokine responsible for amplifying keratinocyte proliferation, neutrophil recruitment, and the release of downstream inflammatory mediators such as IL‐6, IL‐8, and CXCL1. Activated dendritic cells also secrete IL‐23, which promotes the differentiation and expansion of Th17 cells. These Th17 cells release pro‐inflammatory cytokines including IL‐17A, IL‐17F, and IL‐22, which act on keratinocytes to induce hyperproliferation, altered differentiation, and sustained inflammation. In addition, tumor necrosis factor‐alpha (TNF‐α) synergizes with IL‐17A, amplifying cytokine signaling and epidermal dysfunction (Bugaut and Aractingi 2021; Sharma et al. 2022).

The medicinal plants have been regularly used to heal many diseases. It would be advantageous to develop novel alternative therapies for treatment that cause fewer adverse effects (Keseroglu and Gonul 2014). Patients choose herbal medications due to their perceived safety compared to conventional therapies. Herbal medications have diverse structures and multidirectional methods of action, unlike synthetic drugs. Recent studies have demonstrated that plant‐derived compounds act on multiple biological targets simultaneously, offering advantages in complex immune‐inflammatory disorders such as psoriasis, where single‐target drugs often show limited long‐term efficacy (Demir et al. 2025; Özdemir and Demir 2025). There is shred of evidence, which suggest that people living in developed countries use traditional medical practices (Abdala et al. 2012). The global rise in the application of plant‐based medicine can be attributed to its existing ability to control a particular disease claims on their safety for consumption (Perez Gutierrez and Baez 2009). When used, these plant‐based treatments not only show effectiveness but also decrease toxicity.

*E. neriifolia* is a dense, branched shrub of the *Euphorbiaceae* family, ranging from 20 ft (1.8 to 4.5 m) to a cylindrical branch or 5‐angled branches (Kirtikar and Basu 1918). It comprises numerous compounds including alkaloids, carbohydrates, amino acids, flavonoids, saponin, and steroids. The plant is majorly used to treat conditions like psoriasis, eczema, piles, and fistula. It is also used to treat joint pain, inflammation, earache, urinary tract disorder, obesity, high cholesterol, anti‐ulcers, splenomegaly, anemia, kidney and bladder stones, and bloating (Mali and Panchal 2017).

Molecular modeling with docking simulation investigates the relationship between ligands and macro‐molecular domains. Analyzing the most desirable configuration aids in determining the interaction or bonding strength of two molecules using an algorithm for scoring (Musfiroh et al. 2013). Through in silico research, computational chemistry has made it easier to identify innovative, effective drugs with fewer side effects (Paul et al. 2018).

Modern research has identified the IL‐23/IL‐17 cascades, TNF‐α, and Th17 cells as central drivers of disease pathology, making cytokine‐specific targeting a key therapeutic strategy. Although biologics and immunosuppressants provide clinical benefit, long‐term use is often associated with adverse effects, high cost, and loss of efficacy, underscoring the need for safer and more accessible alternatives. It serves as a central effector cytokine in this pathway, which is clinically validated as a therapeutic target for the selective inhibition of IL‐17A. It represents a rational strategy for anti‐psoriatic drug discovery (Ghoreschi et al. 2021). Therefore, the present computational study focuses on the interaction of phytoconstituents from *Euphorbia neriifolia* with IL‐17A, supported by density functional theory (DFT) analysis and in vitro cell‐based bioassay, to explore their potential as natural IL‐17A inhibitors.

## 2 Materials and Methods

### 2.1 Extraction of Phytoconstituents From *Euphorbia neriifolia*

The phytochemical composition of *E. neriifolia* has been extensively described in classical literature, with consistent reports identifying triterpenoids (euphol, cycloartenol), steroids (β‐sitosterol), flavonoids, and diterpenes as predominant constituents (Sultana et al. 2022). Although detailed analytical characterization of the extract (such as LC–MS or GC–MS profiling) was not performed in the present study, the methanolic extraction method employed here is widely recognized as an efficient approach for isolating these documented classes of compounds. Therefore, the expected composition of the extract used in this work aligns with previously published phytochemical investigations of *E. neriifolia* .

The *E. neriifolia* extracts were prepared by using Soxhlet equipment. The thimble was packed with drug that was in coarse powdered form, and methanol served as the solvent that was used. The procedure continued for 24 h. Following uninterrupted extraction, methanol was vaporized on a water bath, and the extract was stored (Extract Yield 5.81%).

### 2.2 Molecular Docking Analysis

Molecular docking studies were carried out on different plant constituents of *E. neriifolia* . Methotrexate and Apremilast have been chosen as standard drugs for molecular interaction analysis. The molecular docking was done using Auto‐Dock Tools 1.5.6. Plant‐based constituents interact with targeted proteins using the PDB: 5HI4. The command prompt was used to determine the docking score. The interaction of receptors and ligands in Discovery‐Studio 2021 produced the docking outcomes.

#### 2.2.1 Preparation of Target Protein

**FIGURE 1:**
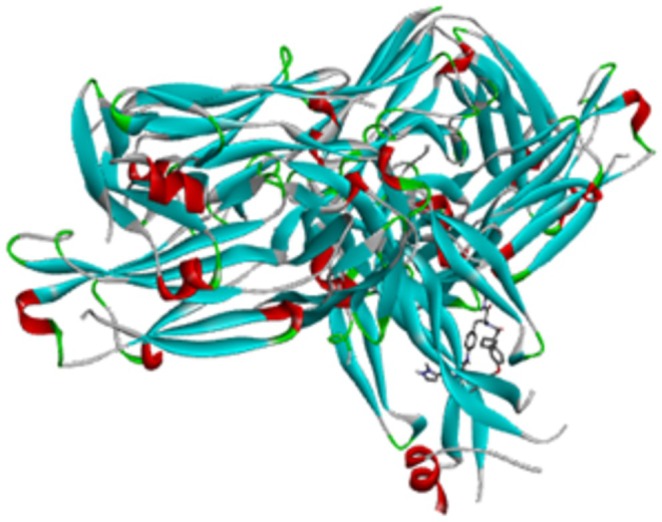
Macrocyclic IL‐17A antagonists' receptor binding protein of psoriasis disease.

A protein target 5hi4 was selected as the target protein and obtained from the RCSB proteins database (www.rcsb.org). Following the removal of the water molecules, Kollman charge and polarized hydrogens were used to support and maintain the protein. The assignment of Kollman charges is particularly important for macromolecules such as cytokines, whereas it enables optimization of the partial atomic charges and stabilizes the protein in a biologically relevant conformation prior to ligand interaction. The addition of polarized hydrogens further refines the hydrogen‐bonding network at the active site, improving docking precision and reducing computational bias (Aydin et al. 2025; Kuzu and Demir 2025). Unneeded binding ligands have been eliminated from the target protein framework (Kumar et al. 2020). The crystalline structure has a 1.80 Å accuracy and contains 132 amino acids in it. Finally, the chosen PDB was transformed into the PDBQT file format using the Auto‐docking tool 1.5.6. Biovia Discovery Studio 2021 was used to prepare its picture, as shown in Figure 1.

#### 2.2.2 Ligand Preparation

**FIGURE 2:**
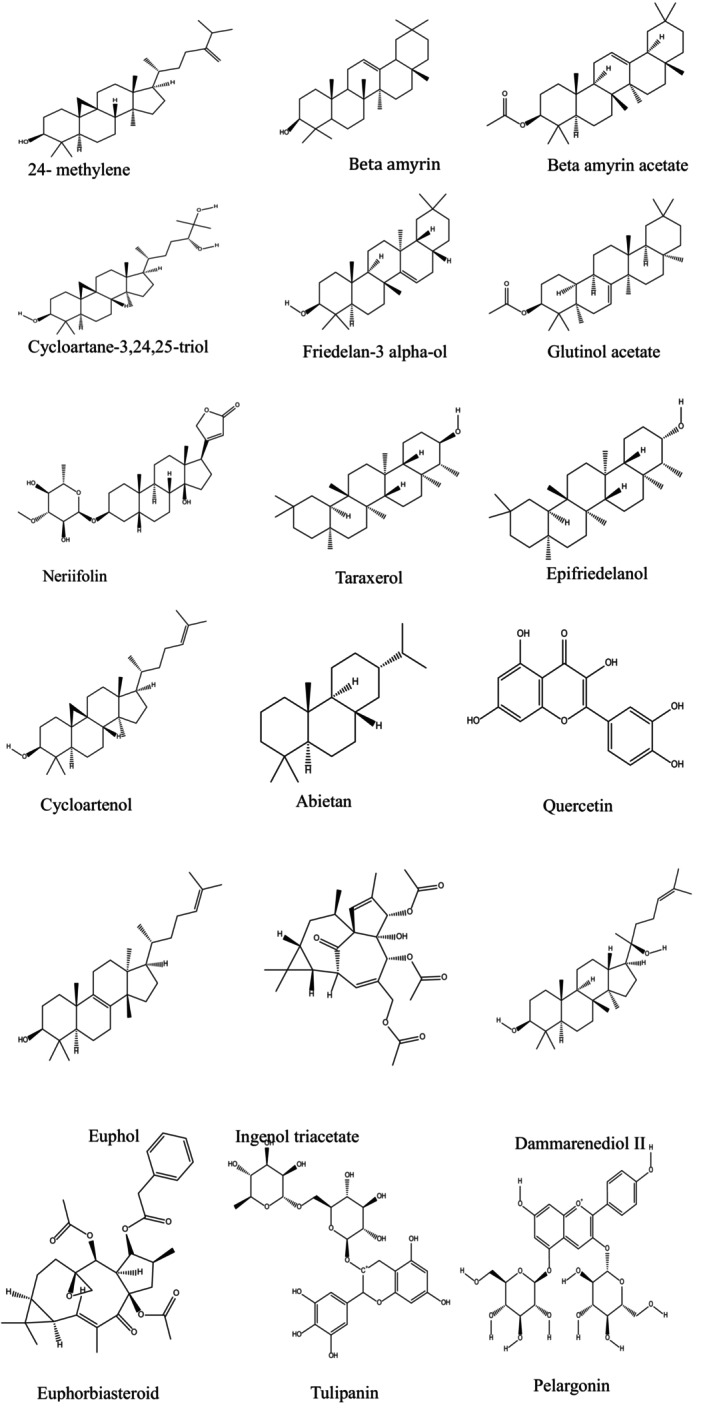
Chemical structures of phytoconstituents from *E. neriifolia* .

The chemical structure of *E. neriifolia* phytoconstituents (Figure 2), including 24‐methylene cycloartanol, Beta amyrin, Beta amyrin acetate, Cycloartane‐3,24,25‐triol, Friedelan‐3 alpha‐ol, Glutinol acetate, Neriifolin, Taraxerol, Epifriedelanol, Cycloartenol, Abietane, Quercetin, Euphol, Ingenol‐triacetate, Dammarenediol‐II, Euphorbiasteroid, Tulipanin, and Pelargonin, were retrieved via the official PubChem website (www.pubchem.org), and Chem Draw Professional 15.0 software was used to create the 2D geometry. The structure was saved with an extension (.cdx), and then a file was saved as (e.g., Pelargonin.cdx). These ligands (phytoconstituents) were subjected to geometry optimization and energy minimization using the MMFF94 force field to obtain the lowest‐energy conformers using Chem3D 15.0 and then saved in extension.pdb. Gasteiger partial charges were assigned, and rotatable bonds were defined to allow flexible docking. The prepared ligands were finally saved in .pdbqt format using Auto‐Dock Tool 1.5.6 for AutoDock‐based analysis (Abul et al. 2025; Farzaliyev et al. 2025).

#### 2.2.3 Molecular Docking Protocols

A semi‐flexible docking approach was employed, where the receptor (IL‐17A) was kept rigid while the ligands were allowed full torsional flexibility. The grid box is created through the ADT “Grid/Grid Box” module to determine the area that can be scanned for docking. The box should contain a protein binding package. In this study, the properties of the box were determined by bringing together 3D‐structured ligands and proteins in the center of the crystal (Table 1).

**TABLE 1:** Grid box employed for molecular docking.

Receptor PDB ID	*X* centre	*Y* centre	*Z* centre	Grid point	Dimensions
5HI4	80.731	−43.501	−45.735	0.375 Å	*X* = 52, *Y* = 38, *Z* = 34 Å

### 2.3 Pre‐ADMET Study

This study was done by using Pre‐Admet online software. ADME stands for absorption, distribution, metabolism, and excretion. In vitro profiles are useful for anticipating the pharmacologic and toxicologic characteristics of candidates for drugs, especially in preliminary phases. To improve toxicity predictions, we used this silico model. Application of such models is beneficial in particular to the customization of drugs and the avoidance of late‐stage breakdowns, which are extremely important since they waste an enormous amount of time as well as funds (Durán‐Iturbide et al. 2020).

The liquid properties of drugs are the effectiveness with which they are broken down in triglycerides or solvents that are not polar. This attribute has a significant impact on the drug's overall AMET properties It is critical for medication absorption across cell membranes. The majority of algorithms (rule 5) for chemical similarity regard a value of lipophilicity in between 0 and 5 as best for drug creation (Waring 2010). The BBB is particularly essential for limiting the entry of drugs into the brain and spinal column (CNS). The current research found that every single compound with the exception of Tulipanin and Pelargonin may diffuse well into the systemic blood circulation. This may be cause for caution about potential CNS‐related adverse effects.

### 2.4 DFT Study

The quantum computational studies of Methotrexate, Taraxerol, Erifriedenalol, and Glutinol Acetate are carried out by DFT methods using B3LYP functionals. The B3LYP functional was chosen because it provides reliable results for geometry optimization and electronic property prediction of organic and bioactive molecules at a reasonable computational cost. The calculations were conducted using Gaussian 16 software, and visualized with Gauss View 6.0 (Frisch et al. 2016). The molecular structures and electronic properties were computed using the 6‐31G (d,p) basis set. The 6‐31G (d,p) basis set, which incorporates polarization functions, enables an elegant representation of molecular orbitals and electron density distribution. After optimization, frequency calculations were performed to ensure the absence of any negative frequencies, which ensures obtained structures converged to a true global minimum. Following the optimization, the study explored the molecular electrostatic potential (MEP), electronic properties, and frontier molecular orbitals (HOMO and LUMO) of the compounds.

### 2.5 In Vitro Study

#### 2.5.1 L929 Bioassay System for TNF‐α

L929, a mouse fibroblast cell line, was cultured in Dulbecco's Modified Eagle Medium (DMEM) supplemented with 10% fetal bovine serum (FBS) and 1% antibiotic solution (penicillin, 100 IU/mL, and streptomycin, 100 μg/mL) at 37°C and 5% CO_2_. The MTT [3‐(4,5‐dimethylthiazol‐2‐yl)‐2,5‐diphenyl tetrazolium bromide] assay was used as a sensitive and reliable method to assess the cellular metabolic activity of the sample on the L929 cell line (Xu et al. 2023).

Cells were cultured at a density of 1 × 10^8^ cells per well in96 well plates at 37°C in a 5.0% CO_2_ atmosphere and allowed to attach for 24 h. The cells were then treated in triplicate with graded concentrations of the test samples at 6.25, 12.5, 25, 50, and 100 μg/mL at 37°C overnight. A 20 μL aliquot of MTT solution was added directly to all the appropriate wells. After 4 h of incubation at 37°C, the media were removed, and the formazan crystals formed as a result of MTT reduction by active cells were dissolved in 80 μL DMSO and incubated for half an hour to dissolve the formazan crystals. The absorbance of each well was read on an ELISA plate reader at 540 nm.

$$\%\text{Cell viability}=\left(\text{Sample}\;\mathrm{OD}/\text{Control}\;\mathrm{OD}\right)\times 100$$

## 3 Results and Discussion

### 3.1 Molecular Docking

To evaluate the binding interaction of the ligands, the interactions with the specific target macromolecules were studied in computational molecular analysis. The structure of macrocyclic IL‐17A antagonists has been linked to conventional medications Methotrexate and Apremilast with Auto Dock Vina 1.5.6, with binding strengths of −9.2 and −9.1 kcal/mol, as shown in Table 2.

**TABLE 2:** Molecular docking score of phytoconstituents with macrocyclic IL‐17A antagonists' targeted proteins.

S. no.	Ligands	Docking value by Auto‐Dock Vina (kcal/mol)
1.	Taraxerol	−10.3
2.	Epifriedelanol	−10.0
3.	Glutinol acetate	−10.0
4.	Cycloartenol	−9.9
5.	24 methylene cycloartenol	−9.7
6.	Friedelan‐3‐alpha‐ol	−9.7
7.	Beta amyrin	−9.6
8.	Cycloartane‐3‐24,25‐triol	−9.5
9.	Beta amyrin acetate	−9.3
10.	Neriifolin	−9.2
11.	Dammarendiol‐II	−9.1
12.	Tulipanin	−9.1
13.	Cycloeucalenol	−9.0
14.	Euphol	−8.9
15.	Euphorbasteroid	−8.9
16.	Pelargonin	−8.8
17.	Quercetin	−8.3
18.	Ingenol triacetate	−7.9
19.	Abietane	−7.7
20.	Methotrexate	−9.2
21.	Apremilast	−9.1

**FIGURE 3:**
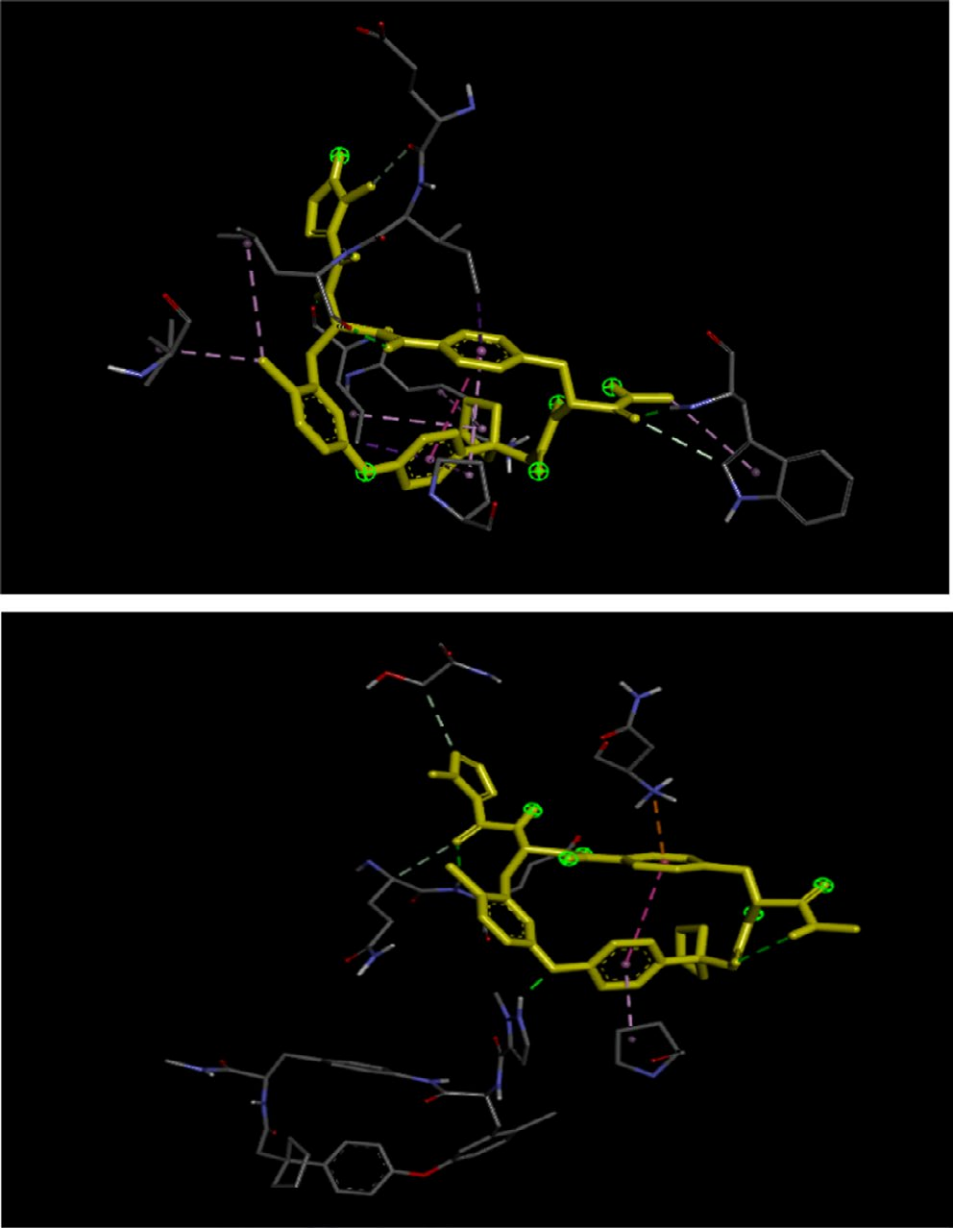
3D interaction poses of 63P co‐crystal ligand with re‐docking.

The docking score between the macrocyclic IL‐17A antagonists and the ligands of chemical components. All have been linked with the energies of binding to measure in “kcal/mol” using AutoDoc Vina that is, −9.7, −9.6, −9.3, −9.5, −9.7, −10.0, −9.2, −10.3, −10.0, −9.9, −7.7, −8.3, −8.9, −7.9, −9.1, −8.9, −9.1, −8.8, accordingly stated in Table 2. The interaction between the standard drug and all the ligands (phytoconstituents) with macrocyclic IL‐17A was summarized in Table 2, and their 2D interaction poses are represented in Figure 3.

Although several ligands in the study share similar core scaffolds, differences in their binding affinities can be attributed to restrained variations in functional groups, steric profiles, and electronic properties. Even minor modifications, such as the presence or absence of a hydroxyl, methoxy, or carbonyl group, can alter hydrogen‐bonding capacity, polarity, or charge distribution, thereby influencing the strength and geometry of interactions within the IL‐17A binding pocket. Additionally, differences in conformational flexibility and the ability of each ligand to adopt an energetically favorable pose contribute to variations in docking scores.

Among all the twelve phytoconstituents, Taraxerol, Glutinol acetate and Epifriedelanol have exhibited significant docking score as standard drugs. Taraxerol exhibited the highest affinity due to the presence of multiple hydrophobic ring systems that complement the non‐polar pocket of IL‐17A, along with two key hydrogen bonds that stabilize the complex. Glutinol acetate demonstrated favorable binding due to its acetyl group, which enhances electronic interaction and enables a strong hydrogen bond with GLU A:95, supported by additional steric contacts. Epifriedelanol showed good affinity mainly due to its polar functional group that forms a stabilizing hydrogen bond with GLN A:93, combined with favorable van der Waals packing against PRO B:37 and TRP B:67.

We have analyzed the binding patterns of all docked phytoconstituents and identified a set of recurring interaction residues within the IL‐17A binding pocket (Table 3), including GLU A:95, TRP B:67, TYR B:62, PRO B:37, and GLN A:94. These residues appeared consistently across several high‐affinity ligands, indicating their role as key contact points for ligand stabilization.

**TABLE 3:** Steric and H‐bond interaction of phytoconstituents with amino acid fragments.

S. no.	Phytoconstituent (ligand)	Hydrogen bond interaction (residues with bond distance Å)	Steric interaction (Van der Waals, π, Alkyl, etc.)
1	Taraxerol	TYR B:62 (2.80 Å), ASP I:8 (2.20 Å)	Alkyl: PRO B:37; VDW: VAL B:65, GLN A:94, TRP B:67, PRO I:6
2	Epifriedelanol	GLN A:93 (2.50 Å)	VDW: PRO B:37, TRP B:67
3	Glutinol acetate	GLU A:95 (2.66 Å)	C–H: GLN A:94 (3.58 Å); VDW: A:63P4000, LYS A:114
4	Cycloartenol	ASP I:8 (1.95 Å)	Unfavorable Donor–Donor: GLN A:94 (1.27 Å); VDW: PRO I:6, TYR B:62, VAL B:117
5	24‐Methylene cycloartenol	GLN A:94 (2.01 Å)	Alkyl: PRO B:63; VDW: A:63P4000, ILE B:66
6	Friedelan‐3‐α‐ol	TRP B:67 (2.76 Å)	VDW: PRO B:37, VAL B:65
7	β‐Amyrin	A:63P4000 (2.43 Å); C–H: A:63P4000 (3.47 Å)	Unfavorable Donor–Donor: GLN A:94 (3.47 Å); VDW: PRO B:63
8	Cycloartane‐3‐24,25‐triol	TYR B:62 (2.44 Å), LEU B:99 (2.42 Å)	π–Sigma: A:63P4000 (3.96 Å); π–Alkyl: TYR A:62 (4.91 Å); Alkyl: LEU B:112 (5.37 Å); VDW: LEU I:9, LEU A:99, LEU A:112, LEU A:97, LEU B:97
9	β‐Amyrin acetate	GLN A:94 (2.36 Å)	VDW: LYS A:114, VAL B:119, PRO B:37, VAL B:117, A:63P4000
10	Neriifolin	GLN A:93 (2.05 Å)	Unfav. Acceptor–Acceptor: TYR B:62 (2.71 Å); Alkyl: PRO B:37; VDW: ASP I:8, GLN A:94, TRP B:51, SER B:41, TYR B:43, VAL B:65, TRP B:65
11	Dammarenidol‐II	TRP B:67 (2.52 Å)	π–Alkyl: TYR B:62 (4.66 Å); VDW: LYS A:114, GLU A:95, A:63P4000, PRO B:37, VAL B:65
12	Tulipanin	LYS A:114 (2.61 Å)	C–H: TRP B:67 (3.70 Å), GLU A:95 (2.97 Å); Unfavorable Donor–Donor: SER B:40 (1.66 Å); π–Alkyl: PRO B:37 (5.21 Å), VAL B:65 (4.91 Å); VDW: A:63P4000, LEU A:97, ASN B:63, ILE B:66
13	Methotrexate	GLU A:95 (3.44 Å), SER B:41 (3.08 Å), TYR B:44 (4.05 Å), TRP B:51 (4.69 Å)	C–H: TRP B:67 (4.69 Å), GLU A:95 (5.51 Å); VDW: VAL B:65, ASN B:36; VAL B:117, GLN A:94, TYR B:43, LEU B:53, ILE B:66, ILE A:92, A:63P4000; π–Alkyl: PRO B:37 (5.89 Å), Val B:119 (4.98 Å)
	Apremilast	GLU A:95 (3.78 Å), TRP B:67 (4.63 Å), ASN B:36 (5.23 Å)	C–H: GLU A:95 (3.82 Å); VDW: ILE B:66, ILE A:96, VAL B:119, GLN A:94, GLN A:93, LEU B:116, SER B:40, A:63P4000; π–Alkyl: PRO B:63 (6.39 Å), LYS A:114 (3.35 Å), LEU A:97 (4.53 Å)

We have also observed that these residues overlap with previously reported IL‐17A inhibitory hotspots described in earlier structural studies, where GLU A:95 and TYR B:62 in particular have been highlighted as critical for antagonist binding and cytokine signaling disruption. This alignment between our docking results and published IL‐17A inhibitor data strengthens the credibility of the predicted binding interactions.

**FIGURE 4:**
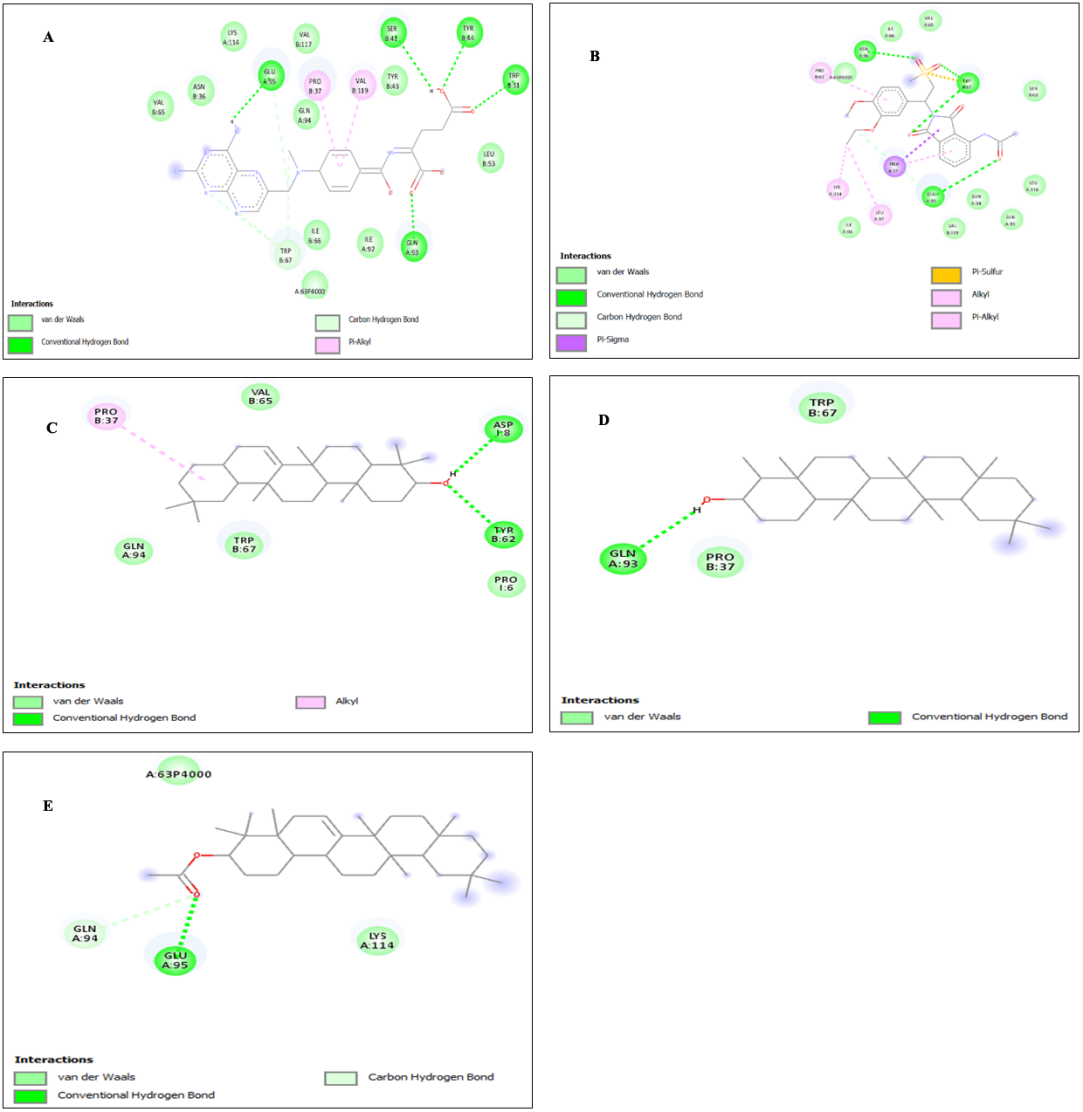
2D interaction poses of phytoconstituents (A‐Methotrexate; B‐Apremilast; C‐Taraxerol; D‐Epifriedelanol; E‐Glutinol acetate).

The steps of molecular docking were validated through the re‐docking of co‐crystal ligand with target protein which was observed to be significant and represented in Figure 4A–E.

### 3.2 Pre‐ADMET Study

Twelve phytoconstituents satisfied all drug‐likeness criteria based on Lipinski's rule of five (Table 5) and were shortlisted using their physicochemical properties (Table 4). Compounds such as 24‐methylene cycloartanol, β‐amyrin, β‐amyrin acetate, cycloartane‐3,24,25‐triol, friedelan‐3‐α‐ol, glutinol acetate, taraxerol, epifriedelanol, cycloartenol, abietane, quercetin, euphol, ingenol triacetate, dammarenediol‐II, and euphorbiasteroid displayed favorable lipophilicity. Their lipophilic nature suggests good membrane permeability, supporting efficient absorption and potentially higher bioavailability.

**TABLE 4:** Physical and chemical traits of generated compounds with good oral bioavailability.

S. no.	Compounds name	Molecular weight	LOG P	HBD^a^	HBA^b^	Molar refractivity	TPSA^c^	%ABS	Lipinski's
1.	24 methylene cycloartenol	440.76	8.73	1	1	135.4	20.23	102.020	0
2.	Beta amyrin	426.73	8.4	1	1	131.73	20.23	102.020	0
3.	Beta amyrin acetate	468.77	8.63	0	2	141.08	26.3	99.926	0
4.	Cycloartane‐3‐24,25‐triol	460.74	6.46	3	3	133.79	60.69	88.0619	0
5.	Cycloartaneol	426.73	8.35	1	1	131.93	20.23	102.020	0
6.	Dammarendiol II	444.74	7.52	2	2	135.63	40.46	95.041	0
7.	Euphol	426.73	8.17	1	1	134.69	20.23	102.020	0
8.	Friedelan‐3‐alpha‐ol	428.75	8.81	1	1	130.91	20.23	102.020	0
9.	Glutinol acetate	468.77	8.63	0	2	141.08	26.3	99.926	0
10.	Neriifolin	534.69	2.59	3	8	140.01	114.68	69.435	1
11.	Taraxerol	412.37	7.93	1	1	127.33	20.23	102.020	0
12.	Epifriedelanol	428.75	8.81	1	1	130.91	20.23	102.020	0
13.	Tulipanin	613.54	−4.08	11	16	—	268.68	16.305	1
14.	Abietane	276.51	6.84	0	0	88.28	0	109.0	1
15.	Cycloeucalenol	426.73	8.19	1	1	131	20.23	102.020	0
16.	Euphorbasteroid	552.66	3.5	0	8	146.29	108.5	71.567	1
17.	Ingenol triacetate	474.55	1.06	1	8	122.29	116.2	68.911	0
18.	Pelargonin	595.53	0.622	10	15	—	250.52	22.570	1
19.	Quercetin	302.24	0.35	5	7	76.5	127.45	65.0297	0
21.	Apremilast	460.50	0.18	1	9	119.65	119.08	67.917	1

^a^ The number of donors of H‐ bonds.

^b^ Number of acceptors of H‐bond.

^c^ Area of the geometrical polar surfaces.

In contrast, compounds such as neriifolin, tulipanin, euphorbasteroid, pelargonin, and apremilast exhibited higher solubility but low lipophilicity, which may reduce their gastrointestinal absorption; however, their solubility profile may pose fewer formulation challenges. A common way to quantify HIA is as a percentage that indicates how much of a specific drug may be absorbed after oral delivery. Drugs with high intestinal absorption in humans (e.g., > 80%) are generally preferred since this indicates that the medication is well absorbed and can reach therapeutic concentrations in the circulation. In the present research, it was discovered that compounds like 24‐methylene cycloartanol, Beta amyrin, Beta amyrin acetate, Cycloartenol, Friedelan‐3 alpha‐ol, Glutinol acetate, Taraxerol, Epifriedelanol had the capacity to be well absorbed into the systemic circulation in the GIT, except for some compounds like Tulipanin and Pelargonin, it is poorly absorbed.

Aqueous solubility refers to a drug's ability to dissolve in water, a key factor influencing oral absorption and overall bioavailability. Drugs with very low solubility may not dissolve adequately in gastrointestinal fluids, resulting in poor absorption and reduced therapeutic effect. In the present study, Tulipanin and Pelargonin showed high numerical values for aqueous solubility, which indicates low actual solubility in water. Hence, both compounds are predicted to exhibit poor aqueous solubility.

Plasma protein binding plays a key role in determining the fraction of a drug that remains bound versus the fraction freely available to exert therapeutic activity. In this study, most compounds showed strong protein‐binding potential, except for Ingenol triacetate, Tulipanin, Pelargonin, Neriifolin, and Apremilast, which exhibited comparatively lower binding. All compounds demonstrated acceptable permeability, with Caco‐2 absorption values falling within the intermediate range, except for Quercetin and Pelargonin, which showed reduced permeability.

Compounds such as taraxerol, epifriedelanol, and glutinol acetate showed high human intestinal absorption (HIA) and complete plasma protein binding (100%). Their aqueous solubility values were low: taraxerol: 0.003, epifriedelanol: 0.001, and glutinol acetate: 0.001—while CACO‐2 cell permeability remained moderate (46.479, 46.844, and 51.090 nm/s, respectively). These properties suggest that the compounds possess good potential for receptor binding, contributing to stronger pharmacological effects relative to the other molecules listed in Table 5. However, their high lipophilicity (logP > 5) may compromise oral bioavailability due to poor solubility and potential issues with membrane transport and metabolic stability. Therefore, structural optimization or advanced formulation approaches (e.g., nano‐carriers, prodrugs, or lipid‐based systems) may be needed to improve their pharmacokinetic performance.

**TABLE 5:** Identified compounds calculated ADME profiles.

Compound name	Blood brain barrier (BBB)	Human intestinal absorption level (HIA)	Aqueous solubility (mg/L)	Caco2 cell^a^ permeability assay	CYP2D6 inhibition	Plasma protein binding (PPB)
24 methylene cycloartenol	21.3383	100.0	0.0004	50.443	NON	100.000
Beta amyrin	21.25	100.0	0.002	46.75	NON	100.000
Beta amyrin acetate	18.5118	100.0	0.001	51.090	NON	100.000
Cycloartane‐3‐24,25‐triol	7.58593	91.826	0.206678	27.907	NON	100.000
Cycloartenol	20.37	100.0	0.001	50.025	NON	100.000
Dammarendiol II	16.0958	94.406	0.032	48.590	NON	100.000
Euphol	21.1674	100.000	0.001	51.100	NON	100.000
Friedelan‐3‐alpha‐ol	22.3207	100.000	0.001	46.844	NON	100.000
Glutinol acetate	18.3039	100.000	0.001	51.090	NON	100.000
Neriifolin	0.287337	90.951	5.384	21.807	NON	87.079
Taraxerol	21.6169	100.000	0.003	46.479	NON	100.000
Epifriedelanol	22.3207	100.000	0.001	46.844	NON	100.000
Tulipanin	0.0284872	1.4516	3685.94	5.0103	NON	70.493
Abietane	17.4862	100.0	0.043	22.201	NON	100.000
Cycloeucalenol	21.3233	100.0	0.001	50.461	NON	100.000
Euphorbasteroid	0.120292	99.469	0.893	34.425	NON	90.005
Ingenol triacetate	0.023007	93.387	26.116	21.645	NON	75.565
Pelargonin	0.0283504	4.997	558.58	3.7468	NON	41.641
Quercetin	0.172765	63.485	96.438	3.412	NON	93.236
Apremilast	0.0963193	98.046	0.742	5.376	NON	83.840

^a^ Caco2‐cell human epithelial colorectal cancer cell lines that are diverse; Permeation of Caco2 cells (nm/s): High (over 70), intermediate (4 tp70), and lowest (below 4); % of gastrointestinal absorption in humans: 70%–100% completely absorbed, 20%–70% fairly absorbed, and 0%–20% badly assimilated; percentage of plasma protein binding: poorly bound (less than 90%), highly bound (greater than 90%).

ADME profiling highlighted key pharmacokinetic differences among the top‐binding phytoconstituents. The compounds showed low predicted BBB permeability, which is appropriate for dermatological agents without CNS targets. CYP450 screening indicated moderate metabolic liability, mainly via CYP3A4, suggesting the need for follow‐up microsomal stability studies. Toxicity predictions revealed no major concerns for mutagenicity or carcinogenicity, though a few compounds showed potential hepatotoxicity alerts. To contextualize these findings, the ADMET results were compared with those of a standard anti‐psoriatic drug (i.e., Apremilast), which displayed comparable safety trends but generally higher metabolic stability. Although the higher lipophilicity of some phytoconstituents may limit oral bioavailability, their favorable safety profile and low CNS penetration support their suitability for topical or localized anti‐psoriatic applications rather than systemic use.

### 3.3 DFT Study

The charge distributions play a key role in determining the reactivity and interactions of the compounds. The electron charge density around the molecule can be understood by the MEP results. Thus, the MEP helps to identify the regions of the molecules that are prone to electrophilic and nucleophilic attack. Negative electrostatic potential was indicated by red and yellow color (high to low intensity), positive potential by blue, and neutral areas by green. The energy gap between the highest occupied molecular orbital (HOMO) and the lowest unoccupied molecular orbital (LUMO), as well as other reactivity parameters like electronegativity, chemical potential, hardness, softness, and electrophilicity, was also calculated from the DFT results. DFT parameters such as HOMO–LUMO energy gap support the predicted chemical reactivity and stability of the lead compounds, indicating their likelihood of forming stable interactions within a biological system.

**FIGURE 5:**
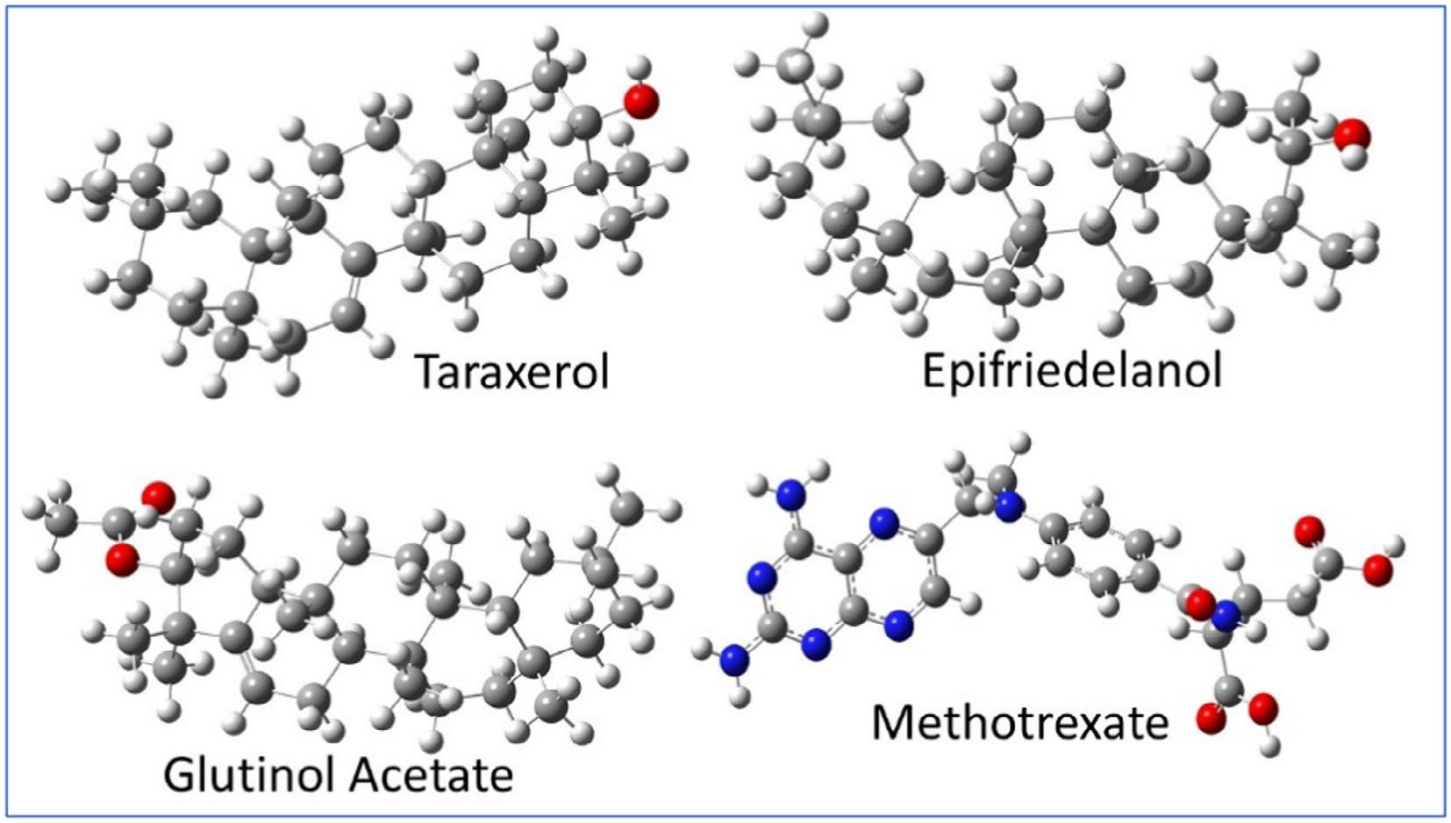
Optimized structure of the ligand molecules obtained through DFT calculations at the B3LYP/6‐31G (d,p) level of theory.

**FIGURE 6:**
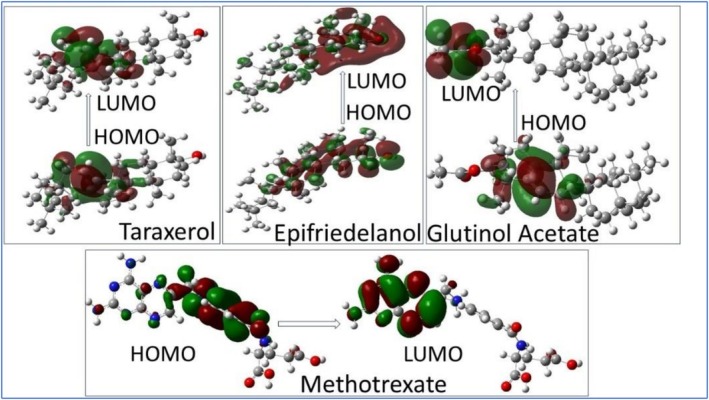
Surface plots of the HOMO and LUMO frontier molecular orbitals, illustrating the distribution and spatial orientation of electronic density for the drug molecules.

The optimized three‐dimensional structures of the drug molecules are shown in Figure 5. All four drug molecules are found to have a C1 symmetry point group. The highest occupied molecular orbital (HOMO) and lowest unoccupied molecular orbital (LUMO) are critical for understanding a molecule's electronic properties, optical behavior, and chemical reactivity. The HOMO‐LUMO energy gap (ΔE) provides valuable information about a molecule's kinetic stability, chemical reactivity, and polarizability. A large ΔE indicates a molecule is hard, with low reactivity and polarizability but high kinetic stability, while a small ΔE points to a molecule with high chemical reactivity and low kinetic stability. The 3D structure of the frontier molecular orbitals is shown in Figure 6. The HOMO‐LUMO energy gap values (Δ*E*) of the drug molecules, along with their chemical hardness and electronegativity, are presented in Table 6. The energy gap (Δ*E*) was found to be the narrowest in the methotrexate molecule among all the compounds studied, which suggests that methotrexate possesses high chemical reactivity. This is further supported by the MEP and Mulliken charge distributions, which reveal highly reactive regions in the molecule.

**TABLE 6:** Energies of frontier MOs, electronegativity, and chemical hardness of the molecules.

	HOMO (eV)	LUMO (eV)	Δ*E* (eV)	Chemical hardness (*η*) (eV)	Electronegativity (*χ*) (eV)
Methotrexate	−5.4883	−2.0719	3.4164	1.7082	3.7801
Glutinol acetate	−6.1751	0.0003	6.1754	3.0877	3.0874
Taraxerol	−6.0423	0.7614	6.8037	3.4018	2.6405
Epifriedelanol	−6.7890	1.6689	8.4578	4.2289	2.5600

**FIGURE 7:**
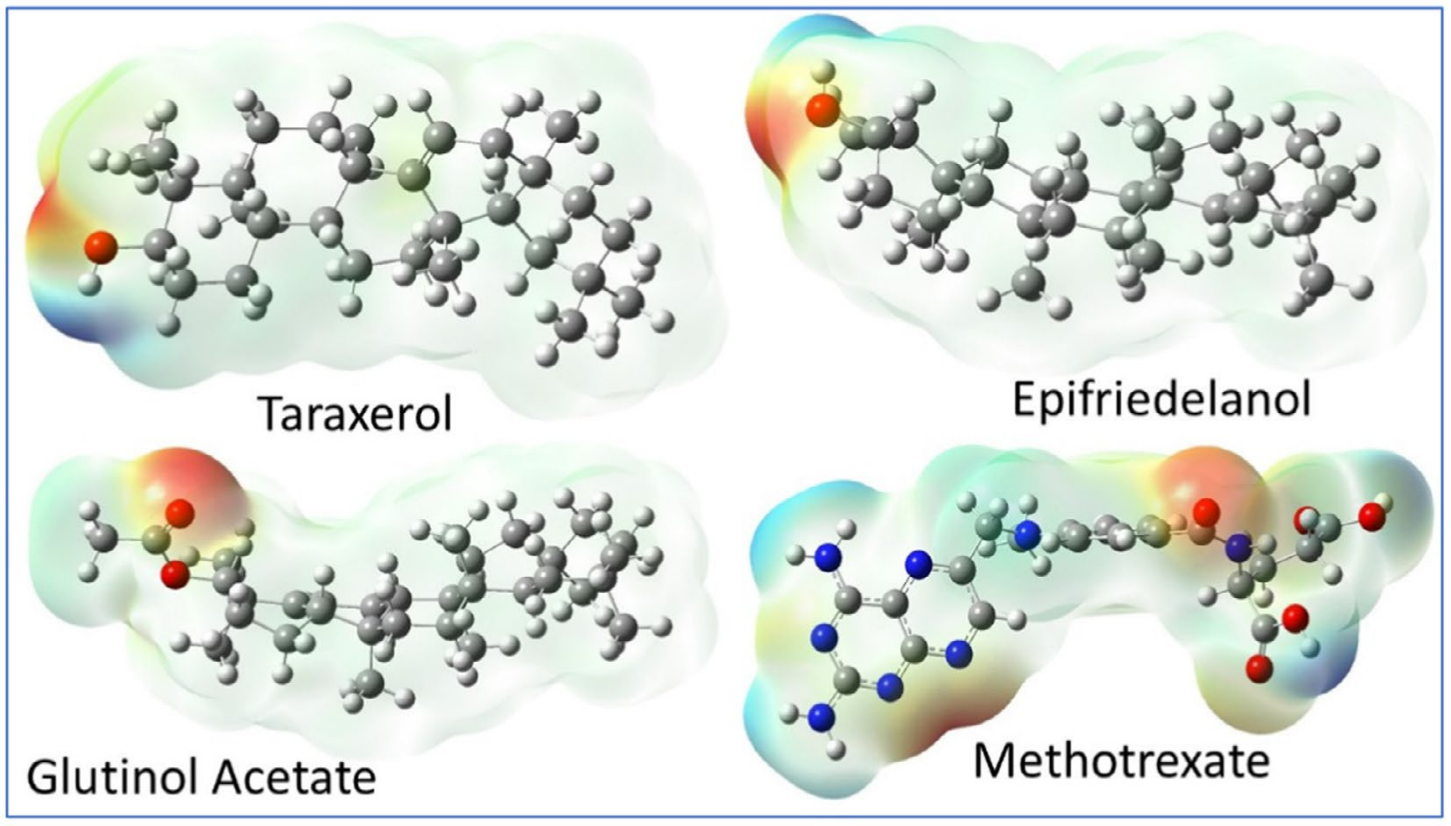
Molecular electrostatic potential (MEP) of the investigated drug molecule. The color coding represents different charge densities, as described in the text. The intensity of the color indicates the magnitude of the charge.

The MEP surfaces of the four drug molecules are displayed in Figure 7. From the MEP images, it is clear that the methotrexate molecules contain several positive (electron‐deficient) and negative (electron‐rich) regions, which can serve as effective active sites. Electron‐rich areas are susceptible to electrophilic attack, while electron‐deficient regions are more prone to nucleophilic attack. The potential sites for electrophilic and nucleophilic attack are much more limited than the other three drug molecules, making them chemically less reactive compared to methotrexate. In other words, it can be said that the proposed three drug molecules may be more selective toward the targeted protein, which can be verified by a docking score.

**FIGURE 8:**
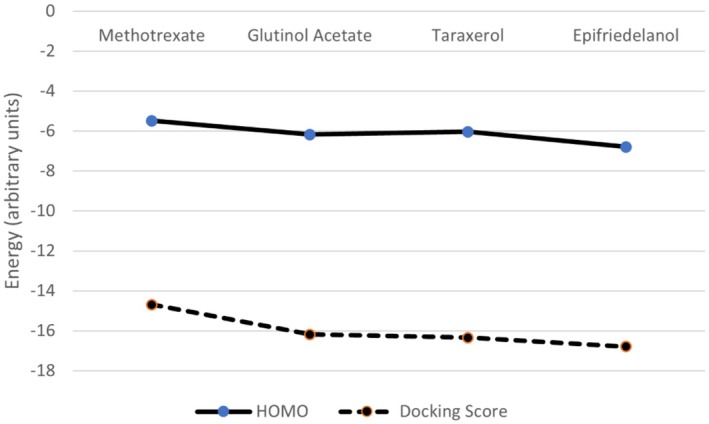
Docking score versus HOMO energy of the four drug molecules.

The HOMO–LUMO gap reflects the intrinsic electronic properties of the isolated molecule, whereas the docking scores depend on intermolecular interactions that are influenced by steric hindrances, hydrogen bonding, hydrophobic effects, etc. The correlation between the calculated HOMO energies and the docking scores is presented in Figure 8, and it was observed that the nucleophilic characteristics (HOMO energies) of these drug molecules are proportional to their docking scores with the target protein. Similar observations were also observed in references (Demir et al. 2023; Yakan et al. 2023).

**FIGURE 9:**
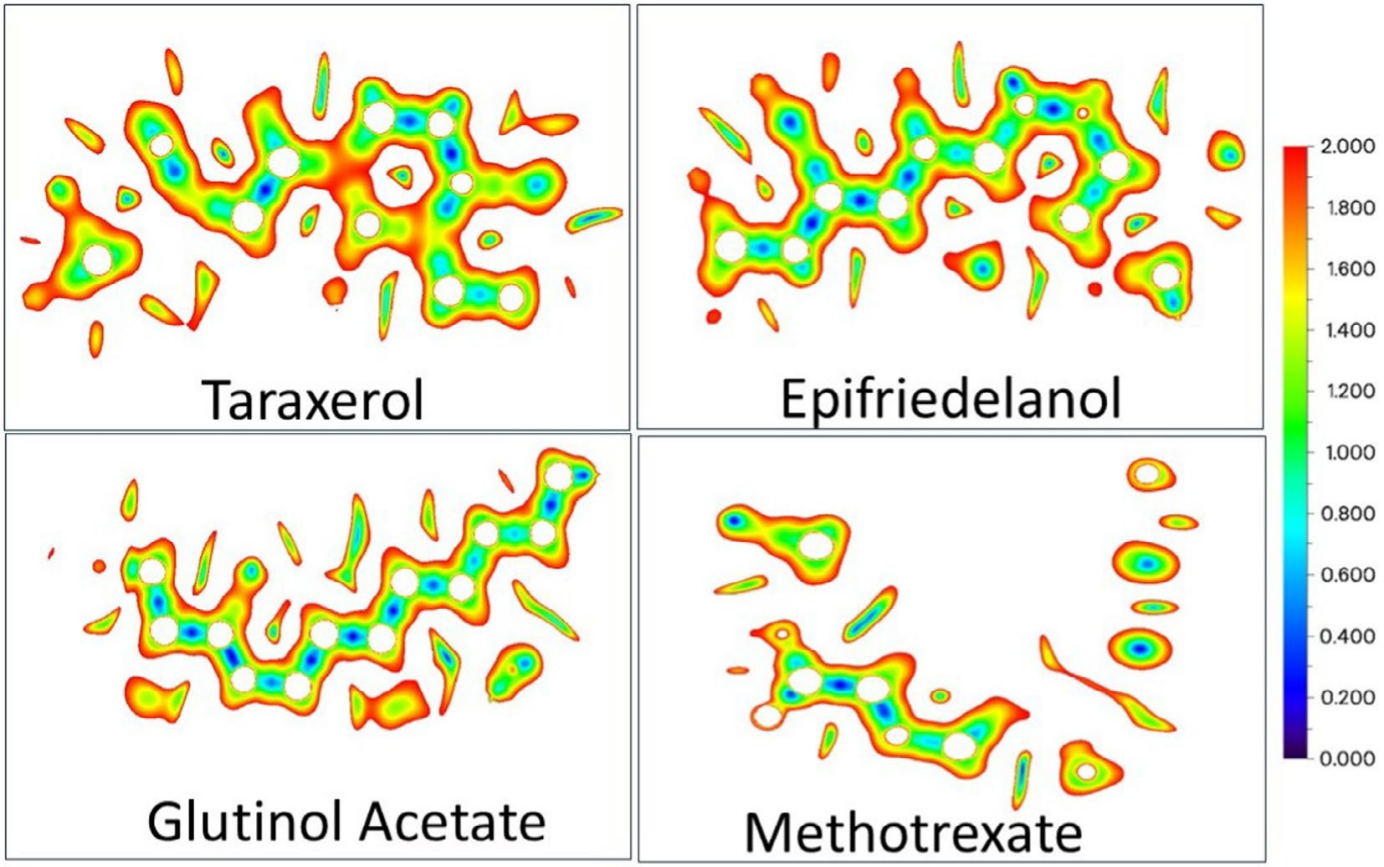
The Interaction Region Indicator (IRI) plot of the studied molecules. The blue–green regions indicate zones of attractive non‐covalent interactions, while red areas correspond to repulsive electronic regions near atomic nuclei.

To have a better understanding of the reactivity of the drug molecules, Interaction Region Indicator (IRI) calculations were carried out from the Gaussian 16 (Frisch et al. 2016) output file using the Multiwfn 3.8 program (Lu 2024; Lu and Chen 2012). The IRI map presented in Figure 9 provides a two‐dimensional visualization of the non‐covalent interaction regions within the molecule. The IRI study identifies the zones associated with weak intermolecular or intramolecular interactions such as hydrogen bonding, π–π stacking, van der Waals forces, or steric repulsion.

In Figure 9, the color scale bar on the right represents the IRI value, ranging from 0.0 (deep blue) to 2.0 (red). The low IRI values (blue–green regions) correspond to weak, attractive non‐covalent interactions and hydrogen bonds. Intermediate IRI regions marked in yellow indicate moderate interactions, such as π–π or C–H···π interactions, and high IRI values (orange–red) represent regions of strong electronic repulsion, which are typically found near nuclear cores or in sterically crowded regions. The white circular zones correspond to the positions of atomic nuclei, where the IRI value is not defined.

The IRI map of Taraxerol reveals a combination of green to yellow regions along the ring structure. It suggests the presence of moderate non‐covalent interactions such as C–H···π or van der Waals contacts. The red contours here indicate steric repulsion within its compact triterpenoid skeleton. A similar trend is observed in Epifriedelanol, which exhibits green‐yellow regions along its carbon chain. These areas indicate a weak dispersion force. The slightly reduced red regions suggest less steric strain in Epifriedelanol compared to Taraxerol. In Glutinol Acetate, more distinct green and yellow areas are observed, particularly around the acetate moiety. It shows the presence of weak interactions such as C–H···O or dipole–dipole contacts. This indicates the coexistence of both hydrophobic dispersion zones and polar interaction sites in the Glutinol Acetate molecule. The widespread low‐IRI regions of the triterpenoids (Taraxerol, Epifriedelanol, Glutinol Acetate) indicate strong potential for hydrogen bonding and hydrophobic contacts with amino acid residues.

In contrast, Methotrexate displays more localized interaction regions consistent with its aromatic and polar structure. The presence of these blue–green patches indicates the potential for favorable intermolecular interactions with the target protein through strong hydrogen bonding, π–π stacking, or dispersion forces. These explain why, despite being the intrinsically reactive molecule, Methotrexate shows a comparable docking score with the studied three triterpenoid molecules.

Overall, the DFT analysis provides a comprehensive understanding of the electronic behavior, reactivity, and interaction potential of the investigated compounds. The HOMO–LUMO energy gaps, MEP surfaces, Mulliken charges, and IRI maps collectively highlight methotrexate as the most intrinsically reactive molecule, with multiple electron‐rich and electron‐deficient regions contributing to its strong interaction potential. Whereas the three triterpenoids exhibit broader regions of weak, non‐covalent interactions and relatively larger energy gaps, suggesting greater stability and more selective reactivity toward the protein environment. These computational insights align well with the docking results, indicating that while methotrexate is inherently more reactive, the triterpenoids possess favorable electronic features that support stable and selective binding to the IL‐17A target.

### 3.4 In Vitro Study

**FIGURE 10:**
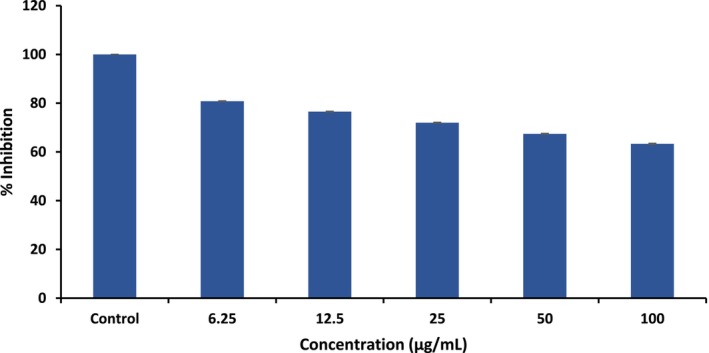
Clustered column graph for percentage inhibition of extract of *Euphorbia neriifolia* .

The anti‐inflammatory effects of *Euphorbia neriifolia* extract were evaluated by using L929 fibroblast bioassay, with a comparative analysis between the control (untreated cells), extract‐treated cells, and methotrexate as a standard drug, shown in Tables 7 and 8. The MTT assay exhibited a significant difference in cell survival rate between the control and extract‐treated group. The untreated L929 cells showed 100% viability, confirming normal fibroblast function. However, the cell viability of *Euphorbia neriifolia* extract remained high (6.25 = 80.81%) at lower concentrations, which indicates the biocompatibility of the extract. At higher concentrations, the cell viability declined (at 12.5 = 76.55%, 25 = 71.55%, 50 = 67.42%, 100 = 63.32%), which indicates potential cytotoxicity or apoptotic effects possibly due to the presence of alkaloids, flavonoids, and triterpenoids in the extract (Figure 10). It could be beneficial for application in psoriasis or inflammatory regulation. The cytotoxicity results showed that the extract exhibited low toxicity at the concentrations effective for anti‐psoriatic activity, indicating that its safety profile aligns with its therapeutic potential.

**TABLE 7:** Observation of L929 bioassay with extract of *Euphorbia neriifolia* .

Conc. (μg/mL)	Control (optic density)	Treated (optic density)	Average viability
6.25	0.789	0.152	80.819 ± 0.124
12.5	0.789	0.186	76.552 ± 0.103
25	0.789	0.22	71.989 ± 0.158
50	0.789	0.258	67.427 ± 0.158
100	0.789	0.289	63.329 ± 0.209

**TABLE 8:** Extract comparison with control.

S. no.	Conc. (μg/mL)	Percentage inhibition	IC50 of extract
1.	Control	100 ± 0.02	168.18 μg/mL
2.	6.25	80.81 ± 0.12	
3.	12.5	76.55 ± 0.1	
4.	25	71.98 ± 0.15	
5.	50	67.42 ± 0.15	
6.	100	63.32 ± 0.2	
7.	Methotrexate		86.2 μg/mL

The increased cytotoxic effect at elevated doses in our study is consistent with previous reports on similar compounds, where a dose‐dependent decline in cell viability has been attributed to enhanced intracellular accumulation and activation of apoptotic pathways (Sriram and Yoganandam 2025; Thomas et al. 2022). However, unlike some earlier studies that demonstrated abrupt cytotoxicity beyond a threshold dose (Karami et al. 2025), our results indicate a more gradual reduction in cell viability, suggesting a potentially wider therapeutic window. This variation may be due to differences in cell line sensitivity, compound purity, or experimental conditions. The alignment and divergence from prior literature have now been clearly highlighted to better position our findings within the existing body of research.

A comparative analysis of the molecular docking and in vitro cytotoxicity results revealed a meaningful correlation between the predicted binding affinity of the phytoconstituents and their biological response in the L929 cell viability assay. Among the screened compounds, Taraxerol, Glutinol acetate, and Epifriedelanol showed the strongest binding affinity toward the IL‐17A active site, characterized by multiple stabilizing interactions such as hydrogen bonding with key residues (e.g., GLU A:95, TYR B:62, GLN A:93) and favorable steric complementarity within the cytokine pocket. These compounds also demonstrated comparatively higher inhibition of keratinocyte proliferation, suggesting that stronger IL‐17A binding may contribute to downstream suppression of inflammatory signaling relevant to psoriasis. Although the in silico and in vitro results do not establish a direct mechanistic link, the observed trend reinforces the potential of these phytoconstituents as lead molecules for further preclinical evaluation. This convergence of computational affinity and experimental cytotoxicity supports a rational prioritization of these compounds for future in vivo anti‐psoriatic studies and possible structural optimization to enhance pharmacological efficacy.

Although L929 fibroblasts were used for preliminary cytotoxicity evaluation, they do not fully represent the psoriatic microenvironment. Future validation will include keratinocyte‐based models (e.g., HaCaT cells) and inflammatory assays involving IL‐17A and TNF‐α to better correlate molecular docking findings with psoriasis‐specific bioactivity.

## 4 Conclusion

Based on the above discussion, the comparative analysis reveals that the phytoconstituents of *Euphorbia neriifolia* exhibit stronger binding affinities toward the receptor protein 5hi4 (macrocyclic IL‐17A antagonist) compared to the standard anti‐psoriatic drugs Methotrexate and Apremilast. The standard drugs demonstrated binding affinities of −9.2 and −9.1 kcal/mol, respectively, whereas the selected phytocompounds like Taraxerol (−10.3), Epifriedelanol (−10.0) and Glutinol acetate (−10.0) displayed higher affinities, suggesting their superior binding potential. These findings indicate that *Euphorbia neriifolia* possesses significant anti‐psoriatic potential. Compounds such as Taraxerol and Epifriedenol have previously been reported to inhibit NF‐κB activation and reduce TNF‐α and IL‐1β release in macrophage models, suggesting their potential to interfere with Th17‐driven inflammatory cascades. If these phytochemicals effectively block IL‐17A binding at the receptor interface, they may suppress keratinocyte hyperproliferation and cytokine overexpression, which are hallmark features of psoriatic lesions. However, further studies are required to validate these results through in vivo models and clinical investigations. Future work will focus on extract purification, dosage optimization, safety profiling, and formulation development to assess the clinical applicability of *E. neriifolia* and its phytoconstituents as potential therapeutic agents for psoriasis management.

Based on the above discussion, the comparative analysis reveals that the phytoconstituents of *Euphorbia neriifolia* exhibit stronger binding affinities toward the receptor protein 5hi4 (macrocyclic IL‐17A antagonist) compared to the standard anti‐psoriatic drugs Methotrexate and Apremilast. The standard drugs demonstrated binding affinities of −9.2 and −9.1 kcal/mol, respectively, whereas the selected phytocompounds such as Taraxerol (−10.3), Epifriedelanol (−10.0), and Glutinol acetate (−10.0) displayed higher affinities, suggesting their superior binding potential. These findings indicate that *E. neriifolia* possesses significant anti‐psoriatic potential. Compounds like Taraxerol and Epifriedelanol have previously been reported to inhibit NF‐κB activation and reduce TNF‐α and IL‐1β release in macrophage models, supporting their possible role in modulating Th17‐driven inflammatory cascades. If these phytochemicals effectively block IL‐17A binding at the receptor interface, they may suppress keratinocyte hyperproliferation and cytokine overexpression, hallmark features of psoriatic lesions. However, as the present study is primarily in silico with limited in vitro validation, these findings should be interpreted with caution. Further studies are required to validate these results through in vivo models and clinical investigations. Future work will focus on addressing these limitations by undertaking extract purification, dosage optimization, comprehensive safety profiling, and formulation development to evaluate the translational potential of *E. neriifolia* and its phytoconstituents for psoriasis management.

## Author Contributions

**Nitin Sharma:** writing – original draft (equal). **Sanjeev Kumar Sahu:** data curation (lead), formal analysis (equal), writing – original draft (equal). **Pankaj Verma:** visualization (lead). **Tanmoy Roy:** formal analysis (equal). **Sourbh Suren Garg:** methodology (supporting), writing – original draft (supporting). **Manish Vyas:** writing – review and editing (equal). **Anil Kumar Sah:** conceptualization – review and editing (equal).

## Funding

The authors have nothing to report.

## Ethics Statement

The authors have nothing to report.

## Conflicts of Interest

The authors declare no conflicts of interest.

## Data Availability

No data was used for the work described in this article.
